# Resensitisation of Methicillin-Resistant *Staphylococcus aureus* to Conventional Antibiotics in the Presence of an Engineered Enzybiotic

**DOI:** 10.3390/pharmaceutics15102511

**Published:** 2023-10-23

**Authors:** Salim Manoharadas, Basel F. Al-Rayes, Mohammed Abdulaziz M. Almuzaini, Yasser Muteq Almohammadi

**Affiliations:** Central Laboratory, College of Science, King Saud University, P.O. Box 2454, Riyadh 11451, Saudi Arabia; 445910355@student.ksu.edu.sa (B.F.A.-R.); 445910144@student.ksu.edu.sa (M.A.M.A.); yasserm@ksu.edu.sa (Y.M.A.)

**Keywords:** methicillin-resistant *Staphylococcus aureus*, enzybiotics, antibiotics, confocal microscopy, bacteriophages

## Abstract

Methicillin-resistant *Staphylococcus aureus* (MRSA) is one of the most dreadful pathogens relevant in community and nosocomial-related infections around the world. Resensitising MRSA to antibiotics, once it became resistant, was a tough choice due to the high adaptability of this bacteria to savage conditions. This study aimed to create a chimeric enzybiotic against MRSA and test its efficiency, either individually or in combination with antibiotics. The novel enzybiotic BAC100 was constructed by fusing the catalytic domain from the bacteriocin BacL_1_ from *Enterococcus faecalis* with the cell-wall-binding domain from protein P17 of *Staphylococcus aureus* bacteriophage ϕ44AHJD. Apart from its partial lone activity, BAC100 was found to resensitise the MRSA strain to traditional antibiotics, including ampicillin and tetracycline. Both drugs were able to reduce live MRSA cells by 85 and 90%, respectively, within 60 min of treatment together with BAC100. However, no significant activity was observed against MRSA when these drugs were tested independently, pointing to the inherent resistance of MRSA against these conventional antibiotics. To our knowledge, this is one of the first instances where an engineered enzybiotic was found to resensitise MRSA to conventional antibiotics. This study will pave the way for the development of similar peptides that can be used together with antibiotics against gruesome pathogens of clinical importance.

## 1. Introduction

*Staphylococcus aureus* is one of the most important human pathogens in the ESKAPE group of Pathogens, which are multidrug-resistant (MDR) and extensively drug-resistant (XDR) [1]. According to the World Health Organization (WHO), ESKAPE pathogens warrant significant attention and are of prime importance in the development of new antibiotics and antibacterial agents [2]. Methicillin-resistant *Staphylococcus aureus* (MRSA) is one of the most predominant pathogens that displays a wide range of antibiotic resistance. MRSA is a leading cause of endocarditis, bacteraemia, tissue infections, and nosocomial infections [3]. MRSA often acquires antibiotic resistance through the insertion of mobile genetic elements (MGE) carrying antibiotic-resistance genes. These insertion sequences, including transposons and plasmids, confer resistance to commonly used clinically relevant drugs, including penicillin (*blaZ*), tetracyclines (*tetK* and *tetL*), and trimethoprim (*dfrA* and *dfrK*) [4].

There have been several attempts to develop alternative therapies against antibiotic-resistant *S. aureus*, either using antibiotics with adjuvants, antimicrobial peptides (AMP), photodynamic therapy, bacteriophage therapy, or the use of nanoparticles and phytochemicals as antibacterial agents [5,6]. For instance, a combination of two antibiotics, Fosfomycin and daptomycin, was found to be effective against *S. aureus* infections [7]. Most of these antibiotic combinations against *S. aureus* have daptomycin as one of the partner drugs. However, daptomycin is the drug of last resort used against critical infections caused by *E. faecium* and *S. aureus* [1]. The potential disadvantages of using a combination therapy of antibiotics cannot be excluded. For instance, the emergence of resistance against monotherapy elicits a narrow spectrum of resistance; however, antibiotic combination with two or more antibiotics selects for a broad spectrum of resistance [8]. Another concern in combination therapy is that one antibiotic used in the therapy can induce a mechanism of resistance against the second antibiotic, which may lead to undesirable effects. In a study where rifampin and colistin were used against *A. baumannii*, no positive effects in the reduction of infection were observed; nevertheless, increased hepatic toxicity was observed [9].

Antimicrobial peptides have shown a wide range of activities against bacterial pathogens. They are short, positively charged oligopeptides produced by bacteria, archaea, fungi, and higher life forms [10]. In contrast to antibiotics other than β-lactams, the AMPs interact with bacterial cell walls and membranes, causing the lysis of bacteria [11]. Since these interactions occur through electrostatic interactions, the emergence of resistance against AMPs is difficult and has never been reported [12]. In addition, AMPs have been shown to be synergistically effective with conventional antibiotics [13,14]. Typically, the AMPs–antibiotics combination allows a longer bacterial pore, preventing pore repair and increasing imbalance in the bacterial cellular osmolarity [13]. The major disadvantage of AMPs is their broad-spectrum activity, similar to antibiotics [15].

Bacteriophage therapy was analysed as an attractive therapy against ESKAPE pathogens, primarily due to high host specificity and the need for low dosage limits [16,17]. A lytic bacteriophage was shown to be very effective against multidrug-resistant *S. aureus* [18]. However, phage therapy also has certain disadvantages, for example, phages can promote horizontal gene transfer in bacteria, contributing to the incorporation of antibiotic-resistance genes [19]. Another concern is the rapid emergence of phage resistance in bacteria. Proper administration of phages is necessary to reach the site of infection. Routinely, phage formulations are either topically applied or orally ingested [20,21]. These disadvantages can be countered if phage-derived endolysins or enzybiotics are used therapeutically instead of intact phage particles. Enzybiotics refer to a new class of antibacterials that are enzyme-based and primarily derived from phage endolysins. Enzybiotics are very specific, safe, and fast-acting antibacterial agents [22]. In addition to canonically derived endolysins, enzybiotics can also be genetically engineered. These proteins have been shown to be very effective against antibiotic-resistant bacterial pathogens and can be used alone or together with conventional antibiotics. In our previous study, we showed the effectiveness of engineered enzybiotics against MRSA and *E. faecalis* [23,24].

The broad objective of this study was to construct a modular peptide, BAC100, and test its efficiency, in combination with traditional antibiotics, against the MRSA strain. BAC100 was constructed by linking the catalytic domain of BacL_1_ with the cell-wall-binding domain of P17, a minor tail fibre protein from phage ϕ44AHJD. BAC100 was found to be partially active against the MRSA strain. The most interesting part of the study was that BAC100 was found to resensitise the MRSA strain to conventional antibiotics when used in combination. Herein, we used two antibiotics, ampicillin and tetracycline. These two antibiotics were chosen because of their different modes of action against bacteria. To the best of our knowledge, this is one of the first instances where chimeric enzybiotics have been shown to resensitise drug-resistant bacteria to traditional antibiotics.

## 2. Materials and Methods

### 2.1. Bacterial Strains and Growth Conditions

Cloning experiments were performed using the bacterial strain *E. coli* XL-1 blue. *E. coli* BL21 (DE3) was used for recombinant protein expression. Both bacterial strains—*E. coli* BL21 (DE3) and *E. coli* XL-1 blue—were gifts from Prof. Dr. Udo Blaesi, MFPL, Vienna, Austria. An MRSA clinical isolate was used to evaluate the activity of the recombinant protein. The MRSA clinical isolate was obtained from King Khaled Hospital in Riyadh, Saudi Arabia.

Luria Bertani (LB) media/agar (0.5% yeast extract, 1% NaCl, 1% peptone; Micromaster, Thane, India), pH 7.0, was used to grow all the bacterial strains. Propagation of bacteria in liquid culture was conducted at 37 °C under shaking conditions (150 rpm) in an orbital shaking incubator (Deluxe automatic orbital shaker, New Delhi, India) unless otherwise mentioned.

### 2.2. Software Used for Protein Structure Prediction

The 3-dimensional structure of the recombinant protein was predicted using the Swiss model program [25]. In this program, both the stoichiometry and the overall structure of the protein complex are inferred by homology modelling based on the amino acid sequence. The default modelling follows five critical workflows such as (a) Input data: the amino acid sequence should be inputted in FASTA format, (b) Template search: based on the input sequence, an evolutionarily related protein structure search is performed against the SWISS-MODEL template library (SMTL), (c) Template selection: after the template search, the resulting models are ranked according to the input template, (d) Model building: a 3D model is automatically generated in the model building stage, (e) Model quality estimation: QMEAN scoring function is used to quantify modelling errors. 

### 2.3. PCR Amplification of Genes

The 987 bp gene fragment of BacL_1_ encoding the catalytic domain (GenBank: AB271686.1) [26] was artificially synthesized (Synbio Technologies, Monmouth Junction, NJ, USA). The primer pair BACFP (5′AATTCGGATCCATGAATTACAGTCAAAAAGCAATCG3′) and BACRP (5′ATTCGGTACCATTCACTGAATCTCCTTTTGAACCAGA3′) was used for the amplification of the 987 bp gene fragment of BacL1. The 300 bp gene fragment encoding the cell-wall-binding domain of P17 (GenBank: NC_004678) [27] was amplified with primers P17300FP (5′CATAGGTACCATCAAAACTGACGCACCATAT3′) and P17300RP (5′CAGGAAGCTTCTATTTTTGATGTTTTGCTACC3′). Amplification of the genes was performed in a thermal cycler (Bio-Rad, Hercules, CA, USA) using a 2× Pfu master mix (G-Biosciences, St. Louis, MO, USA). The gene amplification conditions were as follows: 94 °C for 6 min, 94 °C for 1 min, 63 °C for 30 s, 72 °C for 1.30 min, and 72 °C for 8 min. The amplification reaction proceeded for 35 cycles. Gene amplification was confirmed by agarose gel electrophoresis.

### 2.4. Gene Cloning and Synthesis of Recombinant Protein

The gene amplified with the primer pair BACFP and BACRP was subjected to gel filtration chromatography for purification (Thermo Fisher Scientific, Waltham, MA, USA). The purified PCR-amplified gene fragment was digested with BamHI/KpnI (Thermo Fisher Scientific, Waltham, MA, USA). The expression vector pQE30 was also restricted with BamHI/KpnI. The restricted gene fragment was cloned into pQE30, creating the plasmid pQE-B. Similarly, the P17 gene fragment amplified with primers P17300FP and P17300RP was restricted with KpnI/HindIII and cloned downstream of pQE-B as KpnI/HindIII, creating the plasmid pQE-BAC100. The sequence of the cloned gene was confirmed through gene sequencing. For the synthesis of the recombinant protein, BAC100, the gene cloned under the IPTG-inducible promoter was induced with 0.4 mM IPTG at a culture OD_600_ of 0.45. The cultures were incubated at 16 °C for 18 h under shaking conditions following the induction of protein synthesis. Protein synthesis in the induced culture was confirmed by resolving an aliquot of the culture on a 12% SDS-PAGE gel. The pellet of induced culture obtained following centrifugation (2350× *g*) was stored at −80 °C until further use.

### 2.5. Purification and Refolding of the Synthesized Proteins

Initially, native extraction of the synthesized protein was performed using the standard protocol from Qiagen (Qiagen, St. Louis, MO, USA). Briefly, the centrifuged cell pellet was resuspended in 10 mL of native lysis buffer (10 mM Imidazole, 50 mM NaH_2_PO_4_ 300 mM NaCl, pH 6.5) together with chicken egg white lysozyme (0.05 gm/mL) (Research Lab, Mumbai, Maharashtra, India). The resuspended cell pellet was incubated on ice for 35 min. Sonication (35% power, 5 s pulse, 5 s off; Biosafer, Zhichunli, Beijing, China) of the cell pellet was conducted for 10 min to break open the bacterial cells and release the soluble protein. The sonicated sample was centrifuged (2000× *g*) for 60 min at 4 °C. The supernatant was loaded onto a 12% SDS-PAGE gel and checked for the soluble protein. For denaturation purification of the protein, the sonicated pellet was solubilized in solubilization buffer (0.1% Tris, 48% urea, 0.065% Imidazole, 1.1% NaCl, 1.3% NaH_2_PO_4_, pH 6.5) at 20 °C for 16 h in an orbital shaking incubator. The solubilized pellet was centrifuged at 16 °C for 50 min. Solubilization of the overexpressed protein was evaluated by resolving a small aliquot of the supernatant on a 12% SDS-PAGE gel. For purification of the 6× His-tagged protein, 1.0 mL of Ni-NTA agarose beads was added to the supernatant, followed by incubation for 3 h at room temperature. After incubation, the mixture with Ni-NTA was loaded in an empty column. The protein-bound Ni-NTA beads were washed with washing buffer (0.1% Tris, 48% urea, 0.080% Imidazole, 1.1% NaCl, 1.3% NaH_2_PO_4_, pH 6.5). The Ni-NTA-bound proteins were eluted using elution buffer (48% urea, 1.3% NaH_2_PO_4_, 0.1% Tris, 1.1% NaCl, 0.7% Imidazole, pH 6.5). The purity of the eluted proteins was checked by resolving on a 12% agarose gel. Nanodrop (Bio-Rad, Des Plaines, IL, USA) was used for the estimation of the concentration of the eluted proteins. Refolding of the purified protein was conducted using stepwise dilution to remove urea in buffer A (50 mM Tris-Cl, 9.6 mM NaCl, 0.4 mM KCl, 1 mM EDTA, pH 6.5). Cellular binding and activity of the refolded protein were assessed using binding assay and live–dead staining of bacteria followed by treatment with proteins, with buffer A maintained as the control.

### 2.6. Cell-Binding Activity of BAC100

The cell-wall-binding ability of the recombinant BAC100 protein towards MRSA clinical isolate was evaluated by adding 50 ng of the protein to 1 × 10^7^ MRSA cells in buffer A, pH 6.5. After adding the protein, the mixture was incubated at 37 °C for 10 min. Centrifugation (16,400× *g*) of the mixture was conducted for 2 min following incubation. Unbound proteins in the supernatant were precipitated after centrifugation using TCA (Trichloroacetic acid). Washing of the cell pellet was performed thrice with 1× PBS to remove any transiently bound protein. SDS-PAGE gel loading dye was added to the cell pellet and the sample was incubated at 94 °C for 10 min. The TCA-precipitated protein fraction and the cell pellet were resolved on an SDS-PAGE gel and then subjected to western blot analysis. The gel-resolved proteins were transferred onto a nitrocellulose membrane (Thermo Fisher Scientific, USA) for 20 min at 20 V in a semi-dry western blotting apparatus (Bio-Rad, USA). The protein-transferred nitrocellulose membrane was blocked with 5% milk powder in 1× TBST buffer for 1 h at room temperature under shaking conditions (50 rpm). The blot was washed thrice with 1× TBST buffer at room temperature under shaking conditions (50 rpm). Primary antibody (mouse anti-his antibody, Abclonal, Woburn, MA, USA) at a dilution of 1:3000 (80 ng/mL) was added to the blot and incubated for 16 h at 16 °C. Non-specifically bound residual primary antibody was removed by washing the blot thrice with 1× TBST. Secondary antibody (goat anti-mouse IgG linked to alkaline phosphatase, Elabscience, Houston, TX, USA) at a dilution of 1:12,000 (16.66 ng/mL) was added to the blot and incubated at room temperature for 1 h under shaking conditions (50 rpm). Thorough washing of the blot was conducted thrice with 1× TBST buffer. Ten millilitres of BCIP/NBT (G-Biosciences, St. Louis, MO, USA) substrate was added to develop the blot.

### 2.7. Live/Dead Staining and Confocal Microscopy

BAC100, alone or in combination with antibiotic-treated MRSA cells, was analysed for live and dead cells together with the control according to the protocol described by Manoharadas et al. [28]. Briefly, SYTO9: Propidium iodide (Thermo Fisher Scientific, Waltham, MA, USA) dye at a dilution of 1:1000 (5 μM: 5 nM) was added to the MRSA cells. The mixture was incubated for 10 min at room temperature under dark conditions. The mixture was centrifuged for 2 min and the supernatant was discarded. The pellet was washed thrice with 1× PBS buffer. The stained cells were imaged using a spinning disk confocal microscope (Zeiss, Jena, Germany) after mounting onto a glass slide covered with a cover slip. Live cells were imaged with SYTO9 dye (green cells) at excitation/emission wavelengths of 483/503 nm. Similarly, dead cells were imaged with propidium iodide (red cells) at excitation/emission wavelengths of 535/617 nm. A Rolera Em-C^2^ camera (Zeiss, Jena, Germany) with an oil immersion objective lens specification of 63× was used for image acquisition. The captured images were processed using the Zen lite software (ZEN 3.1 (blue edition), Zeiss, Jena, Germany). The numbers of live (green) and dead (red) cells per frame/picture were counted manually. The experiment was performed in triplicate and the mean value was calculated.

### 2.8. Software Used for Graph Preparation and Statistical Analysis of the Data

The data acquired from image analysis were plotted as graphical representations using Microsoft Excel (2010 version). The mean values and standard deviation values were estimated using Microsoft Excel (2010 version). Deviations in values as evaluated using standard deviation were plotted as error bars. Statistical variations in data and *p*-values were calculated using a one-way ANOVA calculator (https://goodcalculators.com/one-way-anova-calculator/ (accessed on 20 August 2023). Statistical variations of the data in supplementary Figure S1 were calculated using Microsoft Excel (2010 version).

## 3. Results

### 3.1. Construction of BAC100 Protein

**Figure 1:**
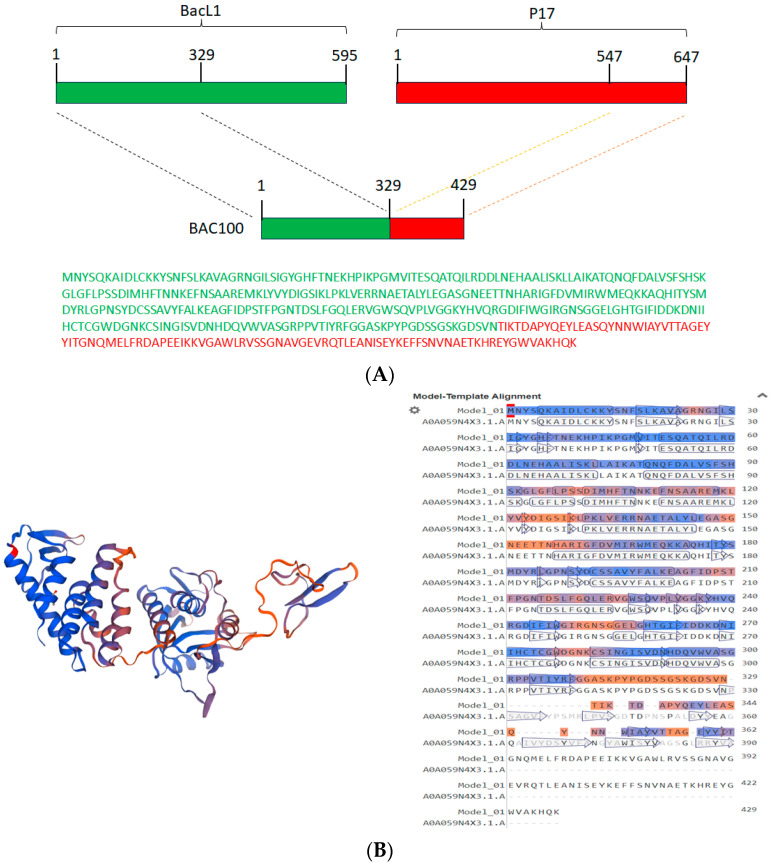
Construction of BAC100 protein against MRSA. (**A**) The catalytic domain of BacL_1_ encompassing 329 amino acids was linked to the cell-wall-binding domain of P17 from phage ϕ44AHJD. (**B**) The Swiss-model-predicted 3-dimensional structure of BAC100 is shown together with the coverage of amino acids used for structure prediction.

The recombinant BAC100 protein was engineered by fusing the 329 amino acid catalytic domain of BacL_1_ with the 100 amino acid cell-wall-binding domain of protein P17 of phage ϕ44AHJD (Figure 1A). The amino acid sequences of both the catalytic domain (green) and the cell-wall-binding domain (red) are shown in Figure 1A. The 3-dimensional structure of BAC100 as predicted by the Swiss model [25] is shown in Figure 1B. The coverage range for the modelling included was amino acids 1-362 and the model was predicted with a sequence identity of 95.03%. The biounit oligomeric state considered for structure prediction was a monomer and the method employed was alphafold v2. The N-terminal catalytic domain of BAC100 comprises coiled structures and the C-terminal region encompasses the cell-wall-binding domain composed primarily of a β-pleated structure.

### 3.2. Synthesis and Purification of BAC100 Protein

**Figure 2:**
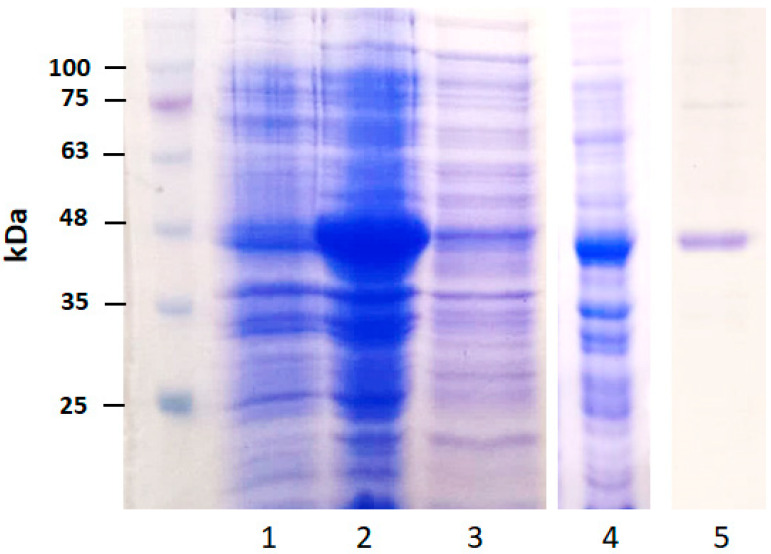
Expression and purification of BAC100. The uninduced culture showed no major synthesis of BAC100 protein (lane 1). The synthesized protein after IPTG induction of the BAC100 gene is depicted in lane 2. The soluble protein fraction as tested on the gel is shown in lane 3. The denatured protein in the presence of 8 M urea is shown in lane 4. The Ni-NTA agarose-purified protein resolved on the gel is shown in lane 5.

BAC100 protein was synthesized by inducing the BAC100 gene in the vector pQE-BAC100 using IPTG. Protein synthesis was allowed to proceed for 18 h at 16 °C following IPTG induction. Synthesis of the BAC100 protein was confirmed by resolving the samples on a 12% denaturing polyacrylamide gel. The uninduced sample loaded on the gel is shown in Figure 2: lane 1. The IPTG-induced sample is presented in Figure 2: lane 2. The synthesized 45 kDa BAC100 protein is shown as a blob of protein. In order to check if the protein is expressed in the native fraction, native purification of the induced pellet was conducted (Figure 2: lane 3). No significant protein of the expected size was observed in the native fraction. Denaturing purification of the synthesized protein was conducted using 8 M urea. The denatured protein is shown in Figure 2: lane 4.

The urea-denatured BAC100 protein was purified using Ni-NTA chromatography and the protein was refolded to its native configuration using stepwise dialysis. The purified and refolded protein was resolved on a 12% PAGE gel to check the purity of the protein. BAC100 was visually judged to be more than 95% pure from the gel (Figure 2: lane 5).

### 3.3. BAC100 Displays Cell Wall Binding to MRSA Clinical Isolates

**Figure 3:**
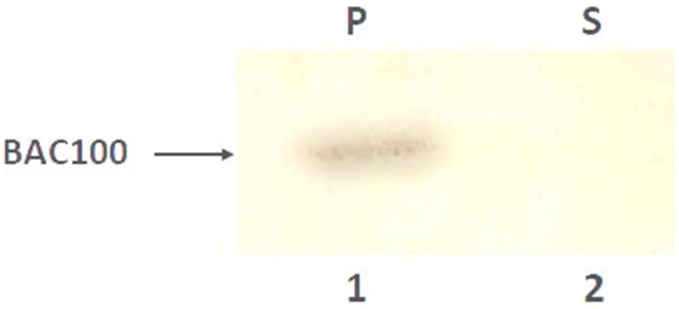
Binding of BAC100 to MRSA. The BAC100 protein was found to bind to the MRSA strain, as detected in the pellet fraction (lane 1). No unbound BAC100 protein was detected in the supernatant fraction (lane 2).

BAC100 protein contains a cell-wall-binding domain from protein P17. In earlier studies, protein P17 and its truncated variants were shown to bind to *S. aureus* bacteria [29,30]. Hence, it was worth testing the cell wall binding ability of the BAC100 protein towards MRSA. As shown in Figure 3, the BAC100 protein was able to bind to the MRSA clinical isolate.

Almost 100% of the input BAC100 protein (50 ng) was bound to the cell pellet (Figure 3: lane 1) and no protein was seen in the unbound supernatant fraction (Figure 3: lane 2). The binding of BAC100 to the MRSA strain was so strong that a stringent washing process did not remove the cell-bound BAC100 protein.

### 3.4. BAC100 Displays Activity against MRSA Clinical Isolate

**Figure 4:**
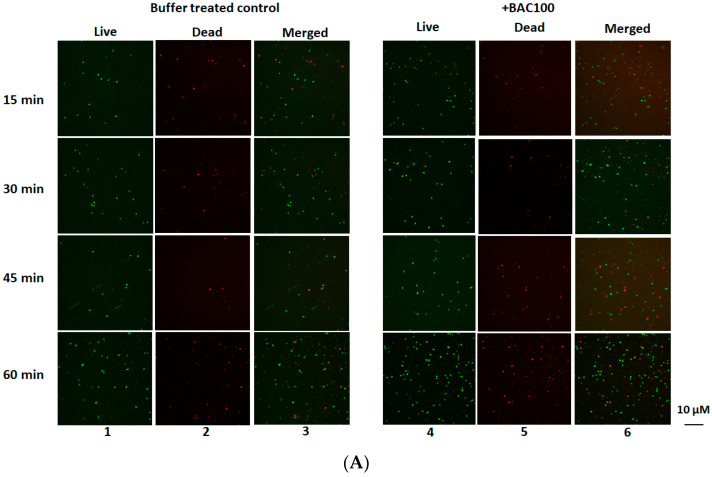

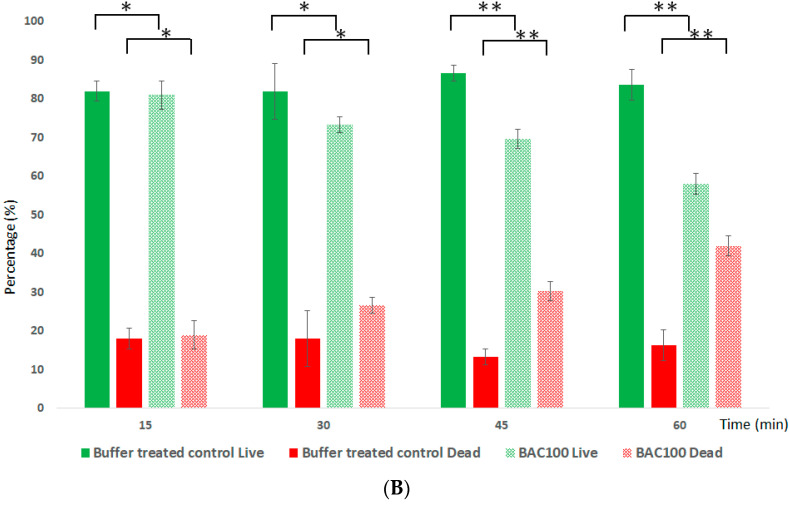
Live/Dead staining of MRSA strain treated with 15,000 ng of BAC100 protein. (**A**) MRSA clinical isolate treated with BAC100. Confocal images of buffer-treated live MRSA taken at various time points are shown in lane 1. Dead cells in the buffer-treated sample taken at different time points are presented in lane 2. The merged image in lane 3 shows both live and dead cells taken at various time points. BAC100-treated live cells (lane 4), dead cells (lane 5), and merged cells (lane 6) taken at different time points. (**B**) Percentage declines in live cells at different time points are shown in a graph. The error bars represent standard deviations. The experiment was performed in triplicate. The statistical test of variance was calculated using one-way ANOVA (* *p* ≥ 0.05; ** *p* ≤ 0.05).

The antibacterial activity of the BAC100 protein against the clinically isolated MRSA strain was tested. A total of 15,000 ng of the purified and refolded protein was added to 1 × 10^7^ CFU per ml of clinical MRSA isolate in buffer A. In one of our earlier studies, we showed that the best activity of a similar chimeric protein with a BacL_1_ catalytic domain was achieved with 15,000 ng of the purified protein [30]. This is why in this study, 15,000 ng of the purified BAC100 protein was added to MRSA. After the addition of the protein, the mixture was incubated at 37 °C for 60 min, with samples analysed for a decrease in the percentage of live cells at 15 min intervals. The BAC100 protein exerted antibacterial activity against MRSA (Figure 4A). However, in contrast to our speculation, no significant activity was observed. A decrease of 45% in live cells was observed at the end of 60 min following incubation with BAC100 (Figure 4B). The decrease in the percentage of live cells increased gradually from 25% at the end of 30 min to 30% at the end of 45 min following incubation (Figure 4B). Statistical significance was calculated using one-way ANOVA (https://goodcalculators.com/one-way-anova-calculator/ (accessed on 20 August 2023). The buffer-treated sample did not show a major degree of variance (* *p* ≥ 0.05) at the time points tested. Also, there was no variance in the data of the buffer-treated control and the protein-treated samples taken 15 min and 30 min following treatment. However, data from the BAC100-treated bacterial samples showed variances at 45 min and 60 min in comparison with the buffer-treated control (** *p* ≤ 0.05).

To test if a higher concentration of BAC100 can cause a reduction in MRSA, 1 × 10^7^ CFU per ml cells were exposed to varying concentrations of BAC100 in vitro. After the addition of BAC100, the mixture was incubated at 37 °C for 16 h. Reduction in the number of viable cells was estimated using spectrophotometric detection and was confirmed using CFU analysis. As shown in Supplementary Figure S1, a maximum of 50 ± 5% reduction in viable cells was only observed with 20 µg/ML of BAC100. The concentration of viable cells was not reduced further, even with a higher concentration of BAC100 tested until 20 µg/ML. The concentration of BAC100 used in combination with antibiotics in this study was 15 µg/M. The 15 µg/ML of BAC100 caused a reduction of 35% in MRSA cells at the end of 16 h following exposure (Figure S1). There was no detectable MIC/MBC for BAC100. 

### 3.5. BAC100 Resensitises MRSA to Ampicillin

**Figure 5:**
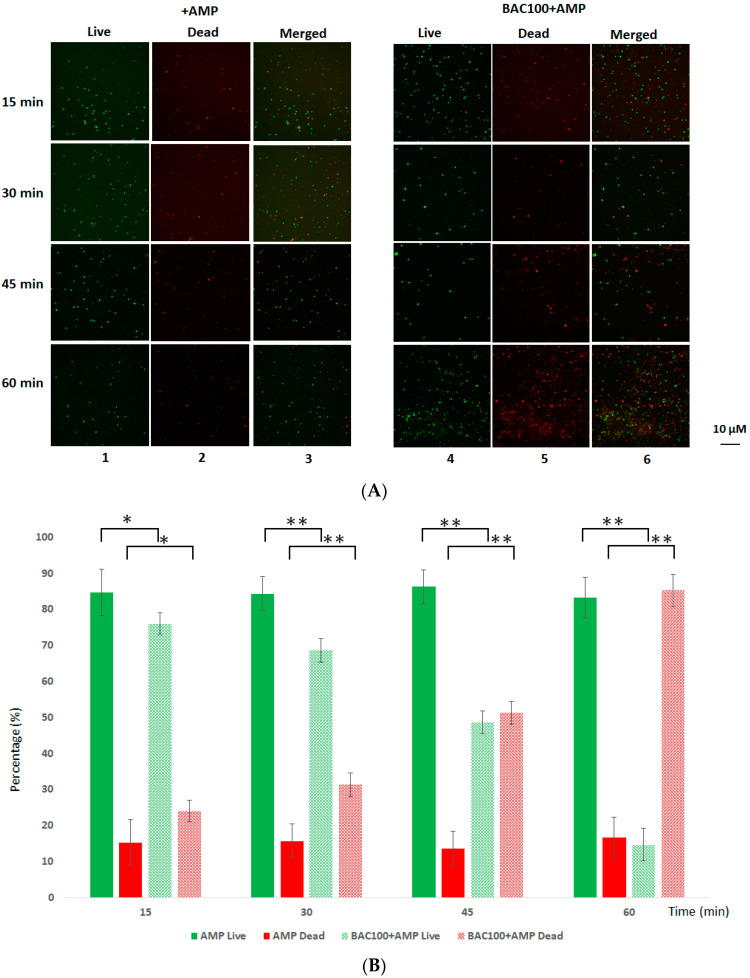
Live/Dead staining of MRSA strain treated with ampicillin and BAC100. (**A**) MRSA clinical isolate was treated with 100 µg/mL of ampicillin. The live cells at various time points of treatment are shown in lane 1. Lane 2 shows dead cells after treatment with ampicillin alone. Live and dead cells as a merged picture are shown in lane 3. Live MRSA cells shown in green in the BAC100 + ampicillin-treated sample taken at various time points are shown in lane 4. Dead MRSA cells following treatment with BAC100 + ampicillin are presented in lane 5. A merged picture showing both live and dead cells (green and red) is presented in lane 6. (**B**) Percentage decline in live cells after treatment with ampicillin alone and after treatment with BAC100 + ampicillin at different time points are presented in a graph. The error bars represent standard deviations. The experiment was performed in triplicate. Statistical test of variance was calculated using one-way ANOVA (* *p* ≥ 0.05; ** *p* ≤ 0.05).

It was earlier shown that MRSA is resistant to the β-lactam antibiotic ampicillin, with the minimum inhibitory concentration being 32 µg/mL [31]. In order to test the efficiency of ampicillin against MRSA clinical isolates in the presence of BAC100, 1 × 10^7^ CFU of MRSA was exposed to 15,000 ng of BAC100 and 10 µg/mL of ampicillin. The mixture was incubated for 60 min at 37 °C. The effect of ampicillin on MRSA in the presence of BAC100 protein was evaluated by observing the reduction in the percentage of live cells in comparison with ampicillin-treated MRSA cells. As seen in Figure 5A and B, no significant reductions in the number of live cells were observed in the ampicillin-treated fraction. However, when both BAC100 and ampicillin were added, a percentage decline in live cells amounting to 30% was seen within 30 min of incubation; this amount increased to 53% within 45 min of incubation. At the end of 60 min of incubation following the addition of BAC100 and ampicillin to MRSA, the percentage of dead cells was approximately 85%, with only 15% of cells in the live fraction (Figure 5B). Interestingly, after 60 min of treatment with ampicillin alone, more than 80% of the cells were found in the live fraction and only 20% were found in the dead fraction. Statistical significance was calculated using one-way ANOVA (https://goodcalculators.com/one-way-anova-calculator/ (accessed on 20 August 2023). The ampicillin-treated sample did not show a major degree of variance (* *p* ≥ 0.05) at the time points tested. However, the BAC100 + Ampicillin-treated sample showed a statistically significant difference in comparison with the control (ampicillin-treated) from 30 min of incubation (** *p* ≥ 0.05).

### 3.6. Tetracycline Actively Annihilates MRSA in the Presence of BAC100

**Figure 6:**
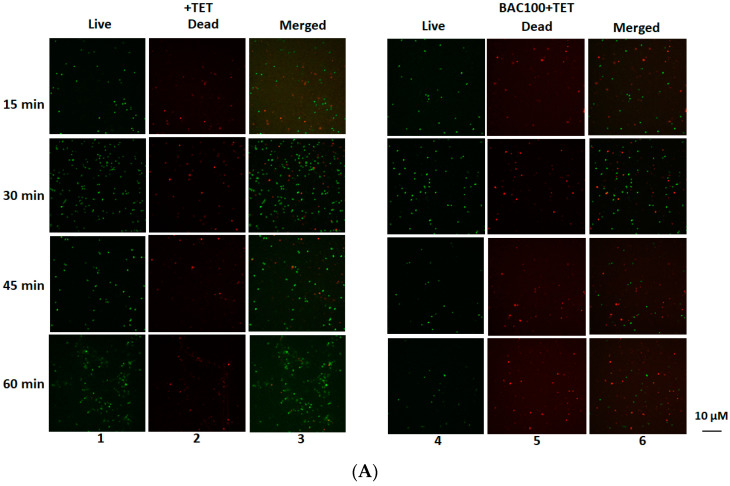

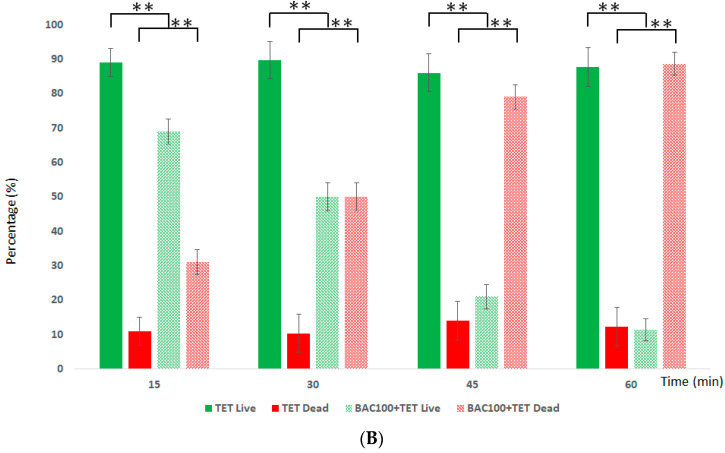
MRSA strain treated with tetracycline and BAC100 analysed by live/dead staining (**A**) MRSA clinical isolate was treated with 10 µg/mL of tetracycline. Lane 1 shows the live cells after treatment with tetracycline. Dead cells after treatment with tetracycline at various time points are shown in lane 2. Lane 3 shows both live and dead cells merged in one picture. BAC100 + tetracycline treatment was able to reduce the live cells within 15 min of treatment. Live cells following treatment with BAC100 + tetracycline are shown in lane 4. Dead MRSA cells (red) following treatment with BAC100 + tetracycline are presented in lane 5. A merged picture showing both live and dead cells (green and red) is shown in lane 6. (**B**) Percentage declines in live cells after treatment with tetracycline alone and BAC100 + tetracycline at different time points are presented in a graph. The error bars represent standard deviations. The experiment was performed in triplicate. Statistical test of variance was calculated using one-way ANOVA (** *p* ≤ 0.05).

Earlier experiments showed that ampicillin, which belongs to the β-lactam class of antibiotics, can act effectively against MRSA in the presence of BAC100. In this further experiment, the effectiveness of tetracycline against MRSA in the presence of BAC100 was evaluated. In earlier studies, it was pointed out that MRSA was resistant to tetracycline, an antibiotic that targets the protein synthesis machinery in bacterial cells [32]. Tetracycline alone (10 µg/mL) was not active in reducing the number of live cells of MRSA, even after 60 min of incubation (Figure 6A: lanes 1, 2, 3). However, in the presence of BAC100 (15,000 ng), tetracycline was able to reduce the live cell concentration from 30 min of incubation (Figure 6A: lanes 4, 5, 6). A reduction of 50% in live cells was observed within 30 min of incubation with tetracycline and BAC100 (Figure 6B). This reduction in live cells increased to 80% by 45 min and 90% by 60 min of incubation (Figure 6B). At the end of 60 min of incubation, only 10% of the live cells were present in the sample. No major decrease in the percentage of live cells was noticed in the tetracycline-only treated control (Figure 6B). Statistical significance was calculated using one-way ANOVA (https://goodcalculators.com/one-way-anova-calculator/ (accessed on 20 August 2023). No major degree of variance was observed in the tetracycline-treated sample (* *p* ≥ 0.05) at the time points tested. However, statistical significance was observed between the BAC100 + tetracycline-treated sample and the control (tetracycline-treated) from 15 min of incubation (** *p* ≥ 0.05).

## 4. Discussion

Methicillin-resistant *Staphylococcus aureus* (MRSA) is an important human pathogen that causes significant health issues globally, impacting patients in both health and community and care settings [33]. The rapid emergence of antimicrobial resistance in MRSA can be largely attributed to the widespread and inappropriate use of antibiotics [34]. The emergence of resistance in *S. aureus* against methicillin dates back to the 1960s, soon after the discovery and widespread use of methicillin [35]. The resistance mechanism in *S. aureus* against methicillin occurs primarily via the expression of the *mecA* gene, which encodes a penicillin-binding protein, PBP2a, that has a low affinity for β-lactam antibiotics [36]. In addition, methicillin resistance was also promoted by the presence of accessory genes such as fem factors [37,38]. Daptomycin, a lipopeptide drug, has been found to be active against MRSA and vancomycin-resistant staphylococcus aureus (VRSA). Daptomycin has also been identified as a partner drug in various antibiotic combinations tested against MRSA, as stated earlier. However, a major concern is that several recent studies have mentioned the emergence of daptomycin-nonsusceptible MRSA strains during the course of treatment [39,40].

Tetracycline is another class of antibiotics, in contrast to β-lactam antibiotics, that targets the protein synthesis machinery in bacteria. Tetracycline resistance in MRSA usually arises from the acquisition of *tet* (tetracycline resistance) and *otr* (oxytetracycline resistance) genes [41]. Resistance to both tetracycline and doxycycline is conferred by the *tet(A)* gene. In community-associated MRSA strains, resistance to tetracycline and doxycycline is derived from the plasmid-borne *tet(K)* gene that codes for an efflux pump [42]. On the other hand, the *tet(M)* gene is a chromosomal insert that encodes elongation factor-like proteins, providing protection for ribosomes from tetracycline [32].

In this study, a chimeric enzybiotic BAC100 was constructed against MRSA. The activity of BAC100 was tested against an MRSA clinical isolate. In contrast to our speculation, BAC100 was only partially active against MRSA. The isoelectric point (pI) of BAC100 protein was calculated to be 6.5. The buffer we used for purification and testing the activity of the protein also had a pH of 6.5. This buffering range rendered no net charge on the protein molecule. This was also one of the primary reasons why the protein was insoluble during overexpression. We had to purify the protein using the denaturation method, with stepwise dialysis, to remove urea and refold it to its native configuration. This could also explain why limited activity against MRSA was observed when the protein was used alone. Interestingly, when used in combination with antibiotics, the sensitivity of MRSA to the antibiotics increased drastically. Two different classes of antibiotics were tested together with BAC100 against MRSA. The first antibiotic tested was ampicillin. Ampicillin is a β-lactam antibiotic classified under aminopenicillins. It binds to penicillin-binding proteins and hampers cell wall synthesis, causing the death of bacteria [43]. MRSA counters this problem by encoding a modified penicillin-binding protein, PBP2a, with a low affinity for binding with β-lactam antibiotics [44]. As expected, the MRSA clinical isolate was resistant to ampicillin, even at the highest concentration of 100 µg/mL. Interestingly, the MRSA strain was resensitised to ampicillin in the presence of BAC100. This feature could be attributed to the localized rupture that is caused by BAC100 in the cell wall structure, allowing access of ampicillin to the otherwise intact peptidoglycan layer. The second antibiotic evaluated for its efficiency against MRSA together with BAC100 was tetracycline. Tetracyclines are broad-spectrum antibiotics that display activity against a wide range of Gram-negative and Gram-positive bacteria. Their mechanism of action is primarily by preventing the attachment of aminoacyl-tRNA to the acceptor site (A) of ribosomes and inhibiting the synthesis of proteins [45]. Staphylococcus aureus and other bacteria counter this by acquiring Tet genes encoding efflux pumps (*Tet A–D* and *TetK*) and ribosomal protection (*TetM*) [46,47]. In this study, it was confirmed that the MRSA clinical isolate was resistant to tetracycline even at the highest working concentration of 10 µg/mL. Interestingly, a notable difference in the number of dead MRSA cells was observed when tetracycline (10 µg/mL) was used together with BAC100, even within 15 min of incubation. As mentioned earlier, localized rupture by BAC100 could allow the entry of a high concentration of tetracycline into the bacterial cells, hence overcoming the efflux pump.

Increasing the therapeutic potentials of conventionally used antibiotics by combining them with chemical or biological molecules was evaluated as an active approach for antibacterial therapy without the emergence of antibiotic resistance [48]. Antimicrobial peptides, phages, and enzybiotics represent a biological class of molecules used in synergistic therapy [49,50,51]. The advantage of engineered enzybiotics is that a domain-switching approach can be employed to create more active proteins that can be used in combination with antibiotics [29]. In this study, we evaluated the potential of an artificially constructed peptide, BAC100, to resensitise MRSA strains to conventional antibiotics. This study will pave the way for the development of similar molecules that can be used in combination with antibiotics, making antibiotic-resistant bacterial strains vulnerable to conventional antibiotics.

## 5. Conclusions

The development of novel antibacterial agents is crucial in the fight against antibiotic-resistant bacteria. There has been extensive research on peptides as antibacterials in the last decade. Even though many antibacterial compounds are in various stages of clinical efficiency studies, significant research is still needed by the scientific community to develop trailblazing compounds against pathogenic bacteria like Methicillin-resistant *Staphylococcus aureus* (MRSA). Herein, we constructed a novel fusion peptide by linking the catalytic domain from the bacteriocin BacL_1_ with the cell-wall-binding domain from protein 17 of phage ϕ44AHJD. The constructed BAC100 protein was partially active against the clinical MRSA strain. Interestingly, BAC100 exhibited significant resensitisation of MRSA to the tested conventional antibiotics, ampicillin and tetracycline, in vitro, when BAC100 and the antibiotics were used in combination. In the next stage of studies, we intend to test the cytotoxicity and immunogenicity of BAC100. We are confident that this study will encourage more research into the resensitisation of antibiotic-resistant bacteria to antibiotics using similar or more potent proteins. 

## Data Availability

The data presented in this study are available on reasonable request from the corresponding author.

## Supplementary Materials

The following supporting information can be downloaded at: https://www.mdpi.com/article/10.3390/pharmaceutics15102511/s1, Figure S1: Viability of MRSA exposed to different concentrations of BAC100.
