# Supplementary material for: Resensitisation of Methicillin-Resistant Staphylococcus aureus to Conventional Antibiotics in the Presence of an Engineered Enzybiotic
Source: Pharmaceutics. 2023 Oct 23;15(10):2511. doi: 10.3390/pharmaceutics15102511 (PMC10610342; doi:10.3390/pharmaceutics15102511)
Supplement: Supplementary file 1 [file pharmaceutics-15-02511-s001.zip › pharmaceutics-2613911-supplementary.pdf]

SUPPLEMENTARY FIGURE

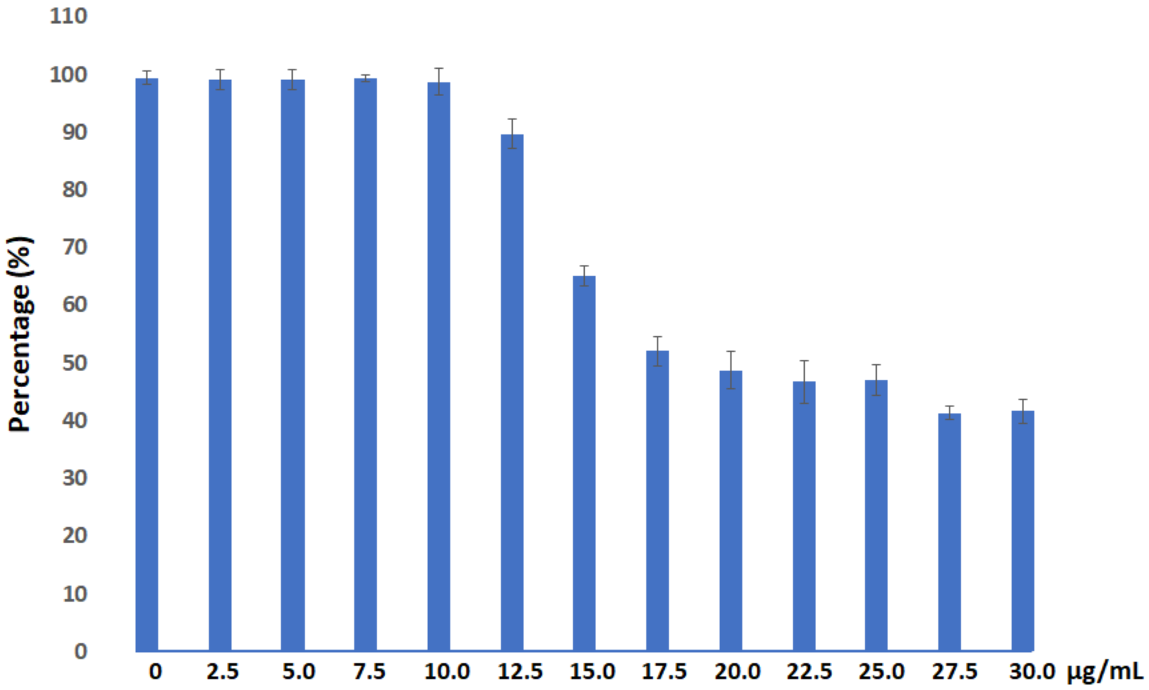

Figure S1. Viability of MRSA exposed to different concentration of BAC100. A 35 % reduction in viable MRSA cells was observed with 15.0 µg/ml of the protein. The viable amount further reduced to 50 % with 15.0 µg/ml of BAC100. No further reduction was observed even when the concentration of BAC100 was increased to 30 µg/ml.
